# Supplementary material for: Expression and Prognostic Value of Chromobox Family Proteins in Esophageal Cancer
Source: Genes (Basel). 2022 Sep 3;13(9):1582. doi: 10.3390/genes13091582 (PMC9498422; doi:10.3390/genes13091582)
Supplement: Supplementary file 1 [file genes-13-01582-s001.zip › genes-1826159-supplementary.pdf]

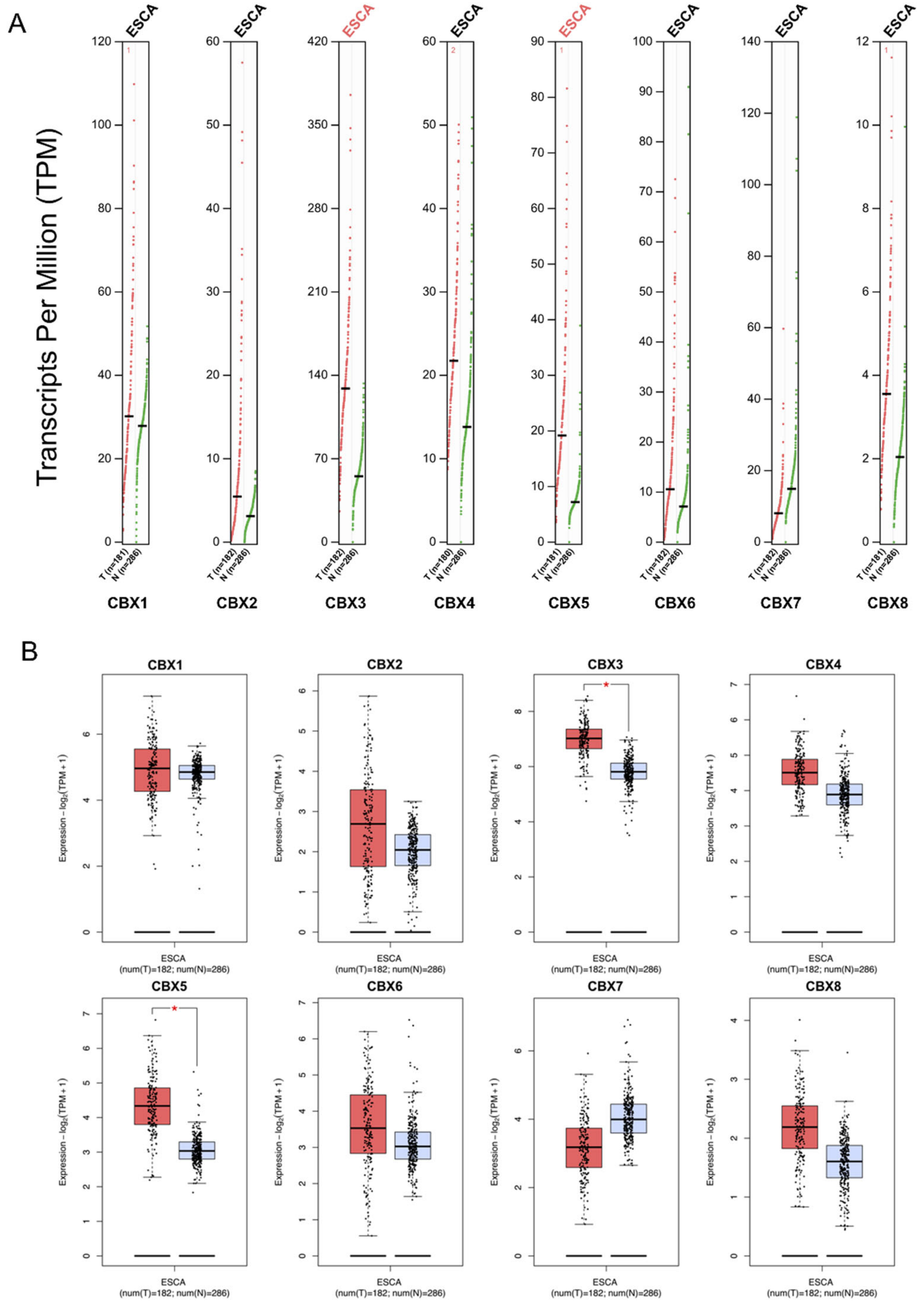

Supplementary Figure S1. Expression of CBX family proteins in EC (GEPIA2). As shown in scatter diagram of CBX mRNA expression (A) and Box plots of CBX mRNA expression(B), CBX3 and CBX5 were significantly overexpressed in EC tissues compared to normal tissues. Red: overexpressed in tumor tissue; green: down-regulated in tumor tissue; black: no significant difference in expression (*p*>0.05). **p* < 0.05 was considered statistically significant.

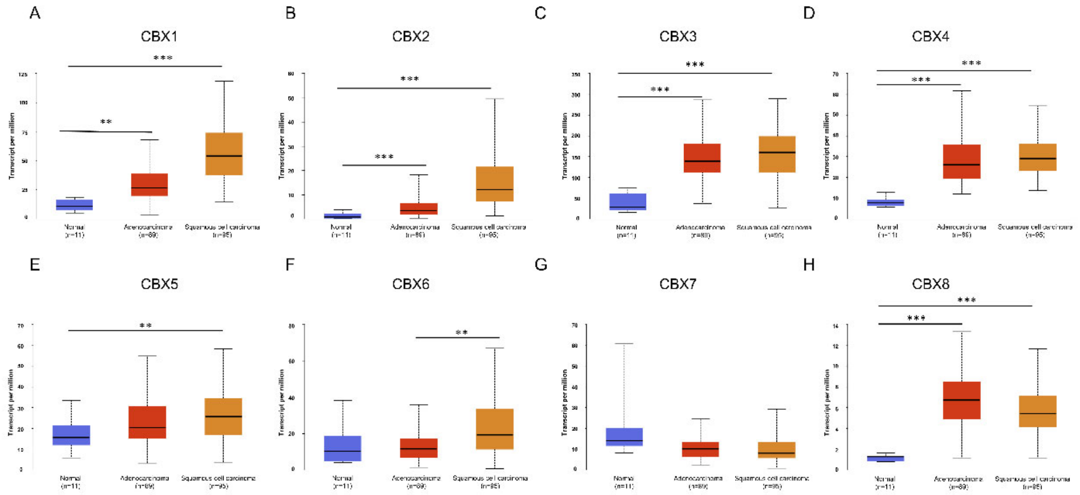

Supplementary Figure S2. Relationships between mRNA expression of CBX family proteins and pathological types in EC patients(A-H). (UALCAN,*p < 0.05,**p < 0.01, ***p < 0.001).

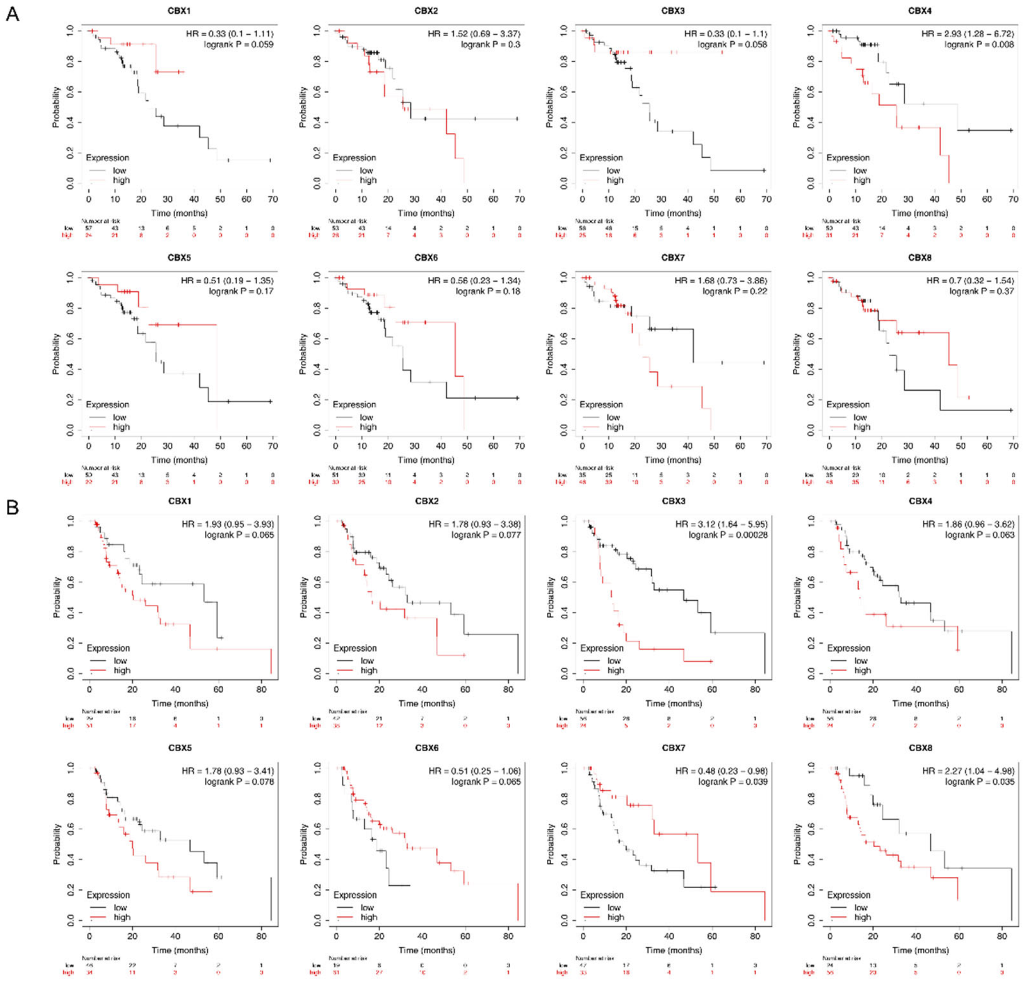

Supplementary Figure S3. Relationships between overall survival (OS) and CBX family proteins

in esophageal squamous cell carcinoma patients (ESCC) (A) and esophageal adenocarcinoma patients (EAC) (B). (Kaplan-Meier plotter, $p < 0.05$ was considered statistically significant).

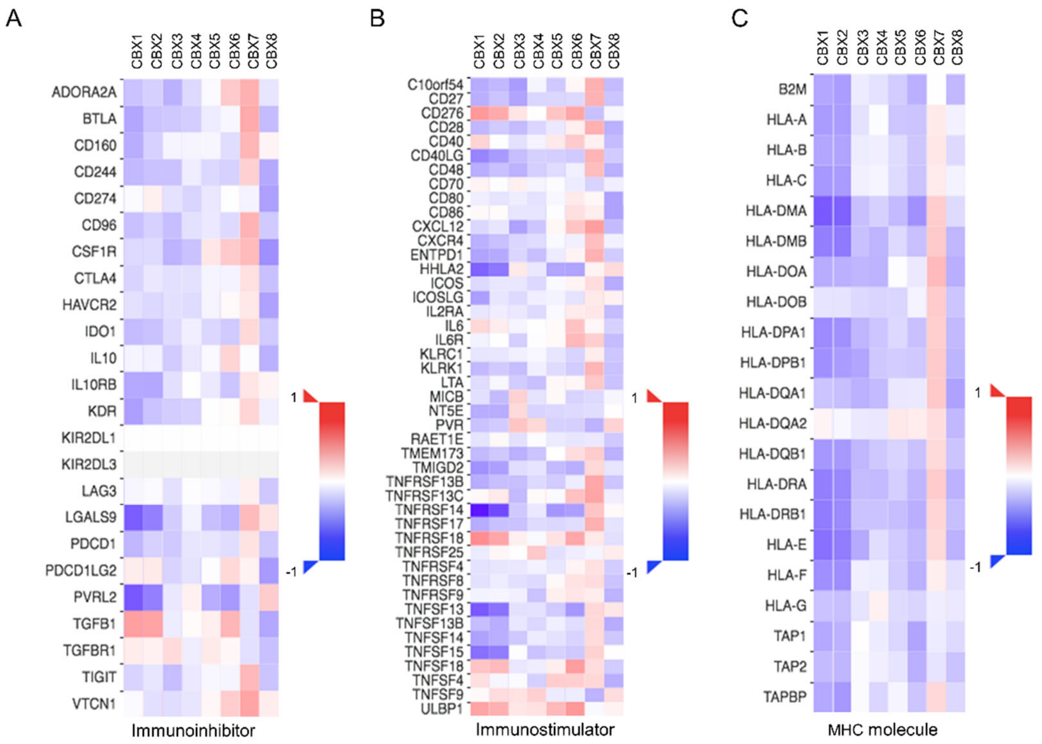

Supplementary Figure S4. Relationships between mRNA expression of CBX family proteins and immunoregulators based on TISIDB database. Correlations between expression level of CBXs and immunoinhibitor (A), immunostimulator (B) and MHC molecule (C). The red, blue and white box indicate positive, negative and no significant relationship, respectively.

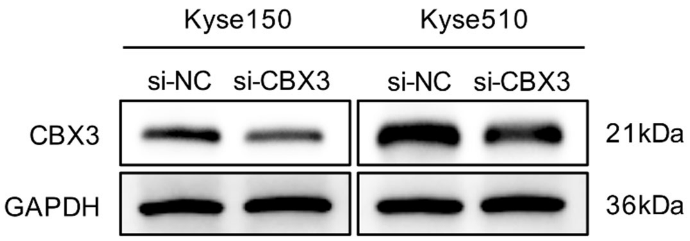

Supplementary Figure S5. The knock-down efficiency of CBX3 siRNA in Kyse150 and Kyse 510 cell lines were confirmed by western blot. GAPDH was the internal reference.
